# OSCILLATOR: A system for analysis of diurnal leaf growth using infrared photography combined with wavelet transformation

**DOI:** 10.1186/1746-4811-8-29

**Published:** 2012-08-07

**Authors:** Ralph Bours, Manickam Muthuraman, Harro Bouwmeester, Alexander van der Krol

**Affiliations:** 1 Laboratory of Plant Physiology, Wageningen University, Droevendaalsesteeg 1, 6708, PB, Wageningen, The Netherlands

**Keywords:** Diurnal leaf movement, Infrared imaging; *Arabidopsis thaliana*, Natural variation, *ERECTA* locus, Wavelet analysis

## Abstract

**Background:**

Quantification of leaf movement is an important tool for characterising the effects of environmental signals and the circadian clock on plant development. Analysis of leaf movement is currently restricted by the attachment of sensors to the plant or dependent upon visible light for time-lapse photography. The study of leaf growth movement rhythms in mature plants under biological relevant conditions, *e.g.* diurnal light and dark conditions, is therefore problematic.

**Results:**

Here we present OSCILLATOR, an affordable system for the analysis of rhythmic leaf growth movement in mature plants. The system contains three modules: (1) Infrared time-lapse imaging of growing mature plants (2) measurement of projected distances between leaf tip and plant apex (leaf tip tracking growth-curves) and (3) extraction of phase, period and amplitude of leaf growth oscillations using wavelet analysis. A proof-of-principle is provided by characterising parameters of rhythmic leaf growth movement of different *Arabidopsis thaliana* accessions as well as of *Petunia hybrida* and *Solanum lycopersicum* plants under diurnal conditions. The amplitude of leaf oscillations correlated to published data on leaf angles, while amplitude and leaf length did not correlate, suggesting a distinct leaf growth profile for each accession. Arabidopsis mutant accession Landsberg *erecta* displayed a late phase (timing of peak oscillation) compared to other accessions and this trait appears unrelated to the *ERECTA* locus.

**Conclusions:**

OSCILLATOR is a low cost and easy to implement system that can accurately and reproducibly quantify rhythmic growth of mature plants for different species under diurnal light/dark cycling.

## Background

The movement displayed by plants has long fascinated people and it is believed that ancient tribes used rhythmic leaf movements to schedule their prayers [1]. The first documented attempt to elucidate whether the rhythm of movement was inherent to the plant or the result of external stimuli was performed by de Mairan in 1729. He observed that the rhythmic leaf movements of his ‘sensitive plant’ (*Mimosa pudica*) continued even in continuous darkness [1,2]. Indeed, leaf movements of many species are controlled by the endogenous circadian clock [3] and in the past decade plant circadian clock research has frequently used leaf movements of *Arabidopsis thaliana* seedlings as a marker of clock output which can be easily compared between different genotypes [4-6].

### Analysis of rhythmic growth in seedlings

Various systems have been described for the analysis of leaf movement in Arabidopsis seedlings [4,6]. These systems are characterised by sequential imaging of seedlings from the side. The position of the cotyledons or the first and second real leaf tip is than recovered from the time series images using for example NKTRACE [7], METAMORPH Software, or custom made programs [8]. Subsequently, the quantified leaf position curves are often analysed using Biological Rhythms Analysis Software System (BRASS). Analysis of the leaf tip plots includes Fast Fourier Transform-NonLinear Least Squares (FFT-NLLS) analysis [9,10]. FFT-NLLS provides the average phase and amplitude of cyclic processes, based on the best fitted sinusoidal curve over multiple days [9] and thus does not capture the daily changes in phase and amplitude upon transition to a different growth condition or during development.

### Analysis of rhythmic growth in mature plants

Methods developed for the analysis of circadian movements or upward leaf reorientation (hyponastic growth) in mature plants include physical attachment of sensors to the plant [11], photoelectric devices developed for measuring leaf movements in space independent of direct contact [12] and strings attached to a rotation resistance transducer glued to the leaf [13]. Time-lapse photography is another commonly used method (*e.g*. [8,14,15]). Imaging from the side makes it difficult to quantify leaf movements of mature plants as the dense whirl of (rosette) leaves may obscure a clear view of single leaves. For this reason, leaves obscuring the petiole base of the tracked leaf need to be removed. This procedure was previously used to quantify hyponastic growth in Arabidopsis and removal of leaves was reported not to influence the movement of the tracked leaves [8]. Moreover, in order to correct for diurnal and circadian effects on leaf movement, the angles of treated and control plants were subtracted in these experiments [8]. Another disadvantage of time-lapse photography is that it commonly depends on standard cameras, which require visible light. It is therefore only suitable for continuous light experiments. To simulate night conditions, non-photosynthetic green light was used to image Arabidopsis leaf growth during the dark period [16]. Similarly infrared imaging has been used to measure the kinetics of Arabidopsis leaf reorientation in response to light quality [15], the response kinetics of Arabidopsis seedlings to ethylene [17] and the growth rate of Arabidopsis hypocotyls in diurnal conditions [18]. Alternatively, images taken at the beginning and end of the day period were used to analyse the diurnal leaf growth and movement of developing Arabidopsis rosette leaves [19]. This approach gives an average growth rate for the light and dark period and can therefore not be used to determine phase, period or amplitude in the leaf growth movement. Here we developed a monitoring system based on infrared (IR) photography called OSCILLATOR. Our system allows continuous, high resolution growth analysis of mature rosette plants, independent of the presence of visible light. It thus enables measurement under biological relevant diurnal photoperiods. We positioned the IR sensitive cameras above the plants. Although imaging from above does not always allow for exact quantification of leaf length due to leaf hyponasty, a top view avoids the problem of rosette leaves obscuring each other. In addition it still allows reliable extraction of leaf movement parameters (phase, period, amplitude) in model species such as Arabidopsis, *Petunia hybrida* (petunia) and *Solanum lycopersicum* L. (tomato) plants. By tracking the movement of the leaf tip of specific leaves over time (typically seven days), growth movement curves were obtained from which phase and amplitude were extracted using wavelet analysis. This processing method allows for reliable measurement of daily phase and amplitude which are convenient parameters to quantify the effect of mutant genes or physiological treatments on growth. To validate the system, we determined the natural variation for diurnal leaf growth movement in several Arabidopsis accessions.

## Results and discussion

**Figure 1:**
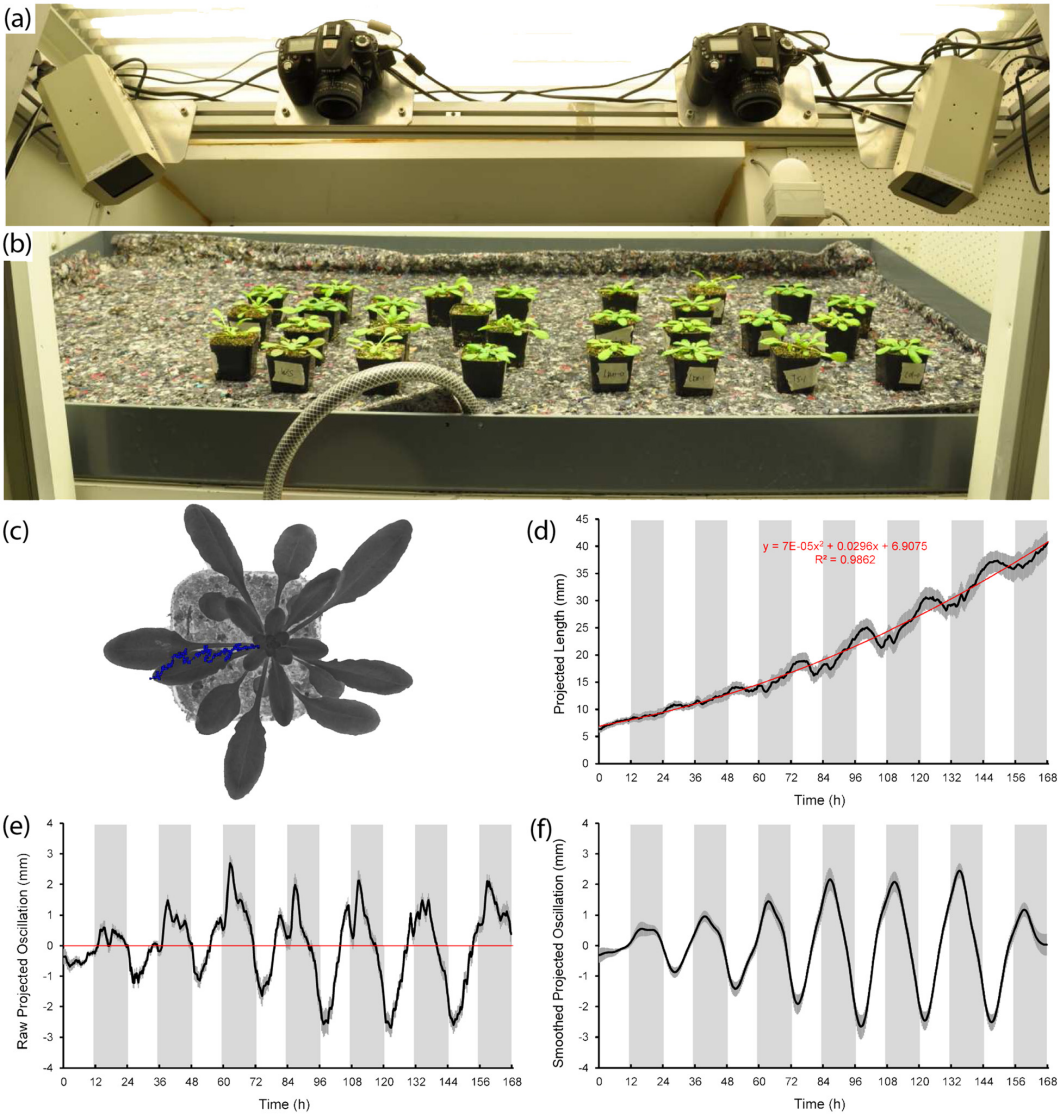
**Experimental setups and the procedure of leaf growth and movement analysis.** (**a**) SLR Cameras are mounted to an aluminium frame inside a growth cabinet, IR illumination is provided from LED lights (far left and far right). (**b**) The camera frame is suspended above a tray containing randomised plants. (**c**) Image J plugins (File S1) allow tracking of the leaf tip throughout a virtual image stack, save the coordinates and project the trajectory. (**d**) The distance in mm from the leaf tip to the rosette centre is calculated, averaged and plotted against time. A best fit 2° polynomial regression line (red) is fitted to individual leaf curves and subtracted from the data. (**e**) The result is the residual from the regression line, here depicted as raw projected oscillations. Note: Originally decreasing distance between tip and centre indicated upward leaf movement. For clarity the residual projected oscillations were inverted to allow maximum upright leaf position to correspond to maximum peak height. (**f**) Harmonic noise is removed from individual leaf growth movement plots using wavelet analysis resulting in smoothed projected leaf oscillation curves. All data represent averages of 10 leaves: For 5 plants, 2 leaves per plant were tracked and the analysis was performed with these 10 leaves ( * n = 10 * ). Because of the high density, the SE’s were plotted for each data point and depicted as shading.

The OSCILLATOR system for continuous analysis of plant growth under continuing diurnal light/dark cycles consists of three modules: (1) data acquisition in the experimental setup (2) image processing and (3) extraction of phase, period and amplitude using wavelet analysis (see Figure 1).

### Data acquisition in the experimental setup

The hardware of the system consists of a climate controlled growth cabinet fitted with two IR LED light units (890 nm) and two modified single-lens reflex (SLR) cameras with the IR filter removed. The cameras are fitted to a sliding frame to allow easy positioning above the plants (Figure 1a). With two cameras per cabinet the full surface area of the growth cabinet could be monitored (Figure 1b) with minimal ‘visual angle-to-object’ effects. Cameras are controlled using NIKON Camera Control software on dedicated laptops. Arabidopsis rosette plants (32 days old) are placed on an irrigation mat which is saturated with tap water every three days. After seven days of acclimation in the growth cabinet images are taken every twenty minutes over a period of up to 16 days.

### Image processing

From the obtained digital image stack of multiple plants, single plant frames were cropped using ImageJ freeware (Figure 1c). For Arabidopsis, for each individual plant, the 11^th^ and 12^th^ real leaves (5 ~ 7 mm long at t = 0) were selected in the first frame and used for measurement of the projected distance between leaf tip to rosette centre throughout the image stack (Figure 1c). This can be achieved in two ways: 1: ImageJ “manual tracking” plugin allows manual tracking of the leaf tip position. 2: ImageJ “Particle tracker [20]” allows measurement of the position of a small dot of inert paint placed on the leaf tip at the start of imaging (this did not affect growth). Both methods provide Microsoft Excel compatible files containing the leaf tip coordinates in pixels (Figure 1c). The rosette centre coordinates were similarly determined in the first frame. For Arabidopsis these remained fixed throughout the experiment. The projected distance in pixels between leaf tip and rosette centre was calculated and converted into millimetres according to a scale marker (placed at average plant height) included in the images. This distance is plotted against time and represents projected leaf length (Figure 1d).

### Extraction of phase, period and amplitude using wavelet analysis

In the projected length curve the vertical rhythmic leaf movements are identified as oscillations in the curve. For the extraction of these oscillations the individualcurves are imported into R freeware and fitted with a best fit 2° polynomial regression line representing average projected growth rate (R^2^ > 0.85) (Figure 1d). The regression curve was subtracted from the leaf tip movement curve, providing a residual oscillation curve. Decreasing distance between tip and centre indicates upward leaf growth movement. Because we want to use the maximum hyponastic leaf position as amarker for the phase of leaf movement, the residual oscillations were inverted to allow maximum upright leaf position to correspond to maximum peak height (Compare Figure 1d and 1e). We confirmed that the peak of oscillations indeed corresponds to the highest leaf angle: For one set of plants the absolute lengths of the tracked leaves were measured daily at the end of the photoperiod (Additional file 1: Figure S1a). Based on the measured projected and absolute leaf lengths, for each day the leaf angle was calculated. A comparison between the smoothed projected leaf tip oscillations and the calculated leaf angle confirmed that peak oscillations match maximal leaf angles (Additional file 1: Figure S1b). On the transition from day to night period a ‘bump’ is observed in the projected leaf growth oscillations. This is caused by a temporary relapse in the upward leaf movement on the light to dark transition (Figure 1e). In order to obtain an objective phase and amplitude of leaf movement, the raw projected oscillation plots of individual leaves were smoothed by removal of harmonic noise using wavelet analysis based on WAVECLOCK [21] (Figure 1f). Wavelet analysis provides an alternative for the commonly used FFT-NLLS method and allows for an accurate day-to-day estimation of phase and amplitude [21]. With wavelet analysis we therefore get an accurate description of adaptations in phase and amplitude throughout development. Smoothed projected oscillation curves of 10 to 12 leaves from 5 to 6 individual plants were averaged and plotted with standard errors for each time point (Figure 1f).

### Characteristics of leaf growth movement in Arabidopsis rosette plants

OSCILLATOR was used to characterise growth of 32 day old Arabidopsis Col-0 rosette plants. For each individual plant, the 11^th^ and 12^th^ real leaves (5 ~ 7 mm long at t = 0) were analysed for seven days. During this period, the projected lengths increased from ~7 to ~ 42 mm. Oscillations initially increased with progressing development, but decreased after ~144 hours and leaves were no longer moving after nine days (Additional file 1: Figure S1c). For characterisation of the phase and amplitude of leaf growth movement in subsequent experiments we therefore chose the developmental window of seven days during which robust oscillations were observed (Additional file 1: Figure S1c). It was previously shown that low levels of IR illumination did not influence seedling development [18]. Nevertheless, we compared projected leaf lengths between plants grown with IR lights (allowing night measurements) and plants grown without IR (day only measurements). Results show an overlap between the IR and non-IR day measurements and final projected leaf length did not differ under these two conditions (Additional file 1: Figure S1a). Therefore, we conclude that also in our system the supplemental IR light does not influence leaf growth movement.

### Natural variation in leaf growth movements

**Figure 2:**
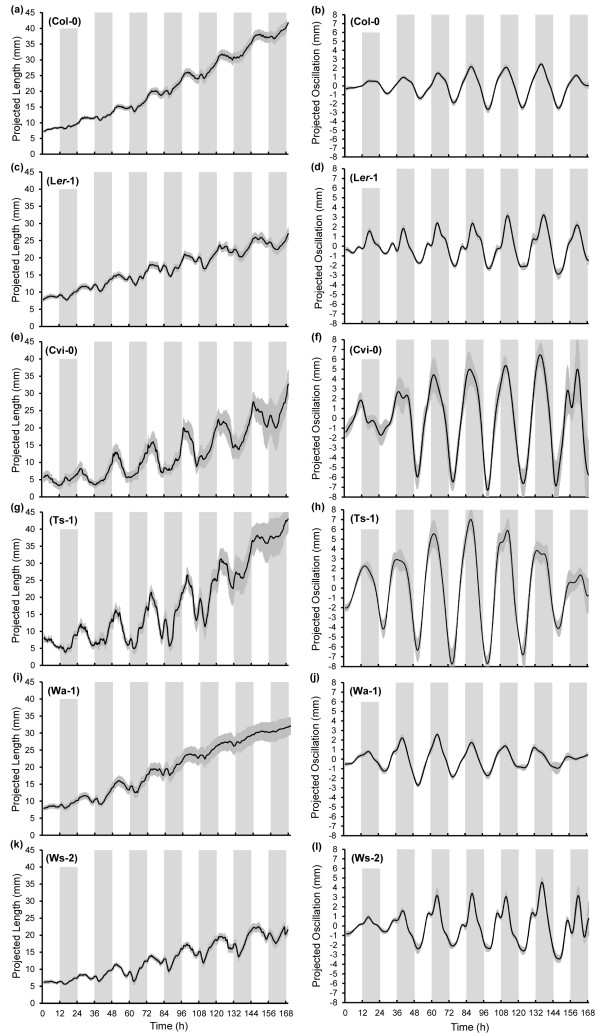
**Natural variation in projected leaf lengths and projected leaf oscillations for selected Arabidopsis accessions.** Projected lengths of selected accessions are depicted in the left column (**a,c,e,g,i,k**) and the inverted and smoothed projected oscillations in the right column (**b,d,f,h,j,l**). For all accessions 2 leaves per plant were analysed and in total 8 leaves (4 plants) were used for analysis ( * n = 8 *) except for Cvi-0 where for one plant only one suitable leaf was tracked, ( * n = 7 *). Error bars represent SE.

**Figure 3:**
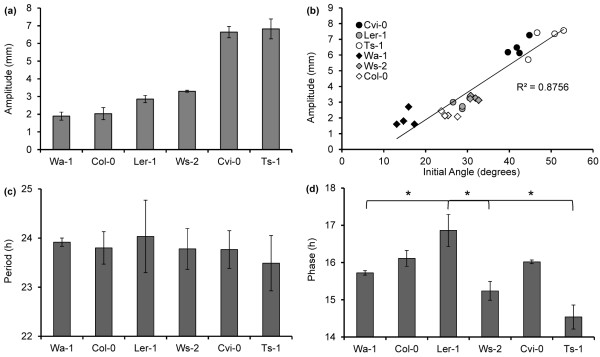
**Natural variation of diurnal leaf growth oscillations in Arabidopsis.** (**a**) Average amplitudes (day 2–6) of each accession. (**b**) correlation between reported angle [22] and measured average amplitude, (**c**) averaged period (day 2–6) of each accession and (**d**) averaged phase of smoothed projected oscillations (day 2 – 6) for the accessions. * n = 8 * leaves, except for Cvi-0 * n = 7 *, from four plants per accession. Error bars represent SE. One-way ANOVA was used to identify significant differences between the accessions. Individual contrasts were then identified in a post-hoc Tukey test. *; *P* < 0.05.

Previously, natural variation in the angle of the petiole of Arabidopsis accessions was determined at a fixed time of the day [22]. In this assay the petiole angle of the different accessions varied between 15.3 degrees in Warschau-1 (Wa-1) to 52.0 degrees in Meloy Ornes [22]. For characterisation of the leaf growth movement we selected six accessions with varying petiole angles, including the common ‘laboratory accessions’ Col-0, L*er-1* and Ws-2 (Additional file 2: Figure S2). For each accession the projected leaf lengths and extracted leaf growth oscillations were determined (Figure 2). Results show distinct differences between leaf movement amplitude in the different accessions. Average amplitudes varied among the dataset from 1.89 ± 0.23 mm (Wa-1) to 6.83 ± 0.56 mm (Ts-1) (Figure 2h,j, Figure 3a). To verify whether variation in leaf petiole angle relates to leaf movement amplitude under diurnal conditions the correlation between reported leaf angles and the amplitudes obtained through OSCILLATOR was determined. Indeed the correlation between amplitude of leaf movement and initial petiole angles [22] was very strong (R^2^ = 0.8756) (Figure 3b). Differences in leaf length in the different accessions could contribute to differences in the measured amplitude. To examine the correlation between leaf length and amplitude these parameters were compared for all accessions. However, no significant correlation between the two was found (Additional file 1: Figure S1d). This indicates that variation in amplitude does not result from variation in leaf length and each accession has a distinct leaf growth profile. The period of leaf movement did not differ significantly and was ~24 hours for all accessions, which may be expected under the diurnal entrainment regime (Figure 3c). The phase of leaf movement varied somewhat in time but was most stable between two and six days where the strongest amplitudes were observed (Additional file 3: Figure S3). The average phase for this period differed between the accessions (P = 0.018). The phase of *Ler-1* (16.9 hours) was significantly later than that of Ts-1 (14.5 hr., P = 0.016), Ws-2 (15.2 hr., P = 0.039) and Wa-1 (15.7 hr., P = 0.048) (Figure 3d). 

### The late phase of L*er*-1 leaf oscillations appears unrelated to the *ERECTA* locus

**Figure 4:**
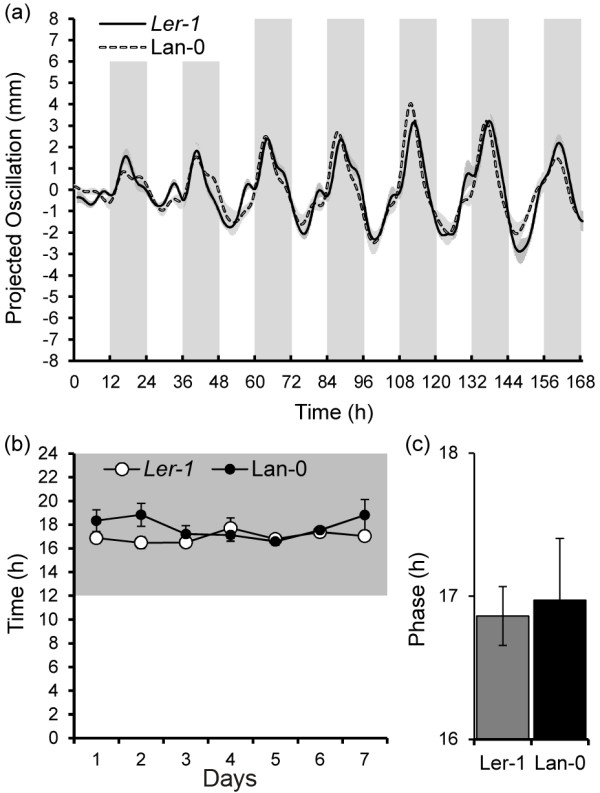
**The* ERECTA *locus does not determine phase of leaf oscillations.** (**a**) Comparison of smoothed projected oscillations between L*er-1* and Lan-0. (**b**) Timing of peak oscillations (phase) depicted per period, grey area indicates night. (**c**) No significant differences were observed for the average phase (between day 2 and day 6) of L*er-1* and Lan-0. Error bars represent SE ( * n * =8).

We investigated whether the late phase of the L*er-1* relates to the null allele of the *ERECTA* (*ER*) locus in this accession. *ER* encodes a leucine-rich repeat receptor-like Ser/Thr kinase, and L*er* carries a missense mutation within the conserved region of the kinase domain [23]. Therefore, *ER* activity differs between Landsberg *erecta* and the original Landsberg wild-type line (Lan-0) from which L*er* was isolated [24]. *ER* has previously been reported to control ethylene induced leaf hyponasty [25,26]. However, it was not investigated whether *ER* affects the phase of leaf movement. Leaf oscillations of L*er-1* were compared to those of Lan-0 (Figure 4a) and results show that the observed late phase of L*er-1* under diurnal light and temperature cycles is also present in Lan-0 (Figure 4b-c). This indicates that in both accessions genetic variation independent of *ER* is responsible for this phenotype. 

### Leaf growth movement in petunia and tomato plants

**Figure 5:**
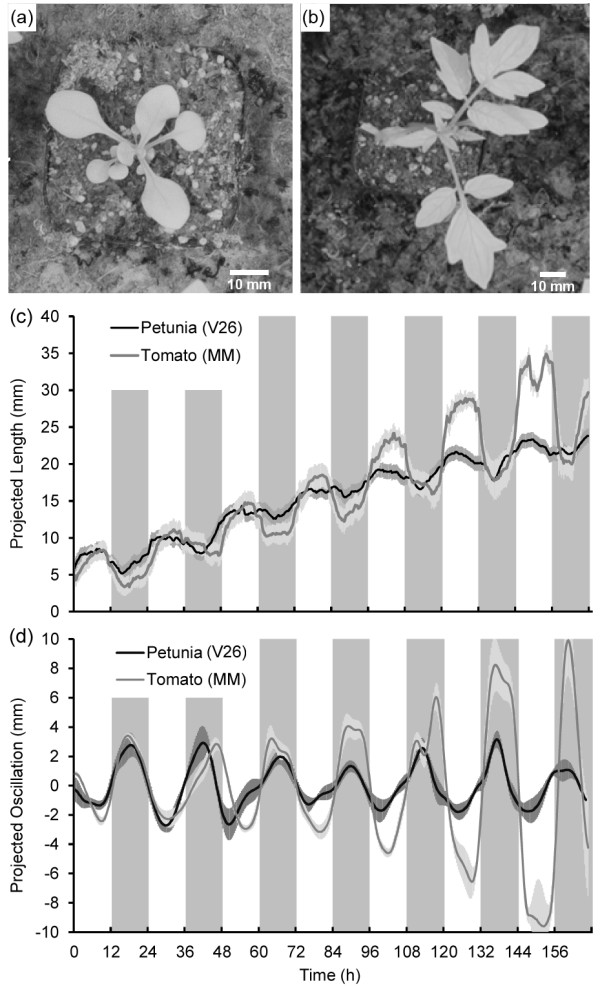
**OSCILLATOR can be used for different species.** (**a-b**) thirty-two day old petunia (**a**) and tomato (**b**) plants at the start of imaging. (**c**) Projected lengths were measured using OSCILLATOR. (**d**) From the projected lengths the projected oscillations were extracted, inversed and smoothed. Error bars represent SE, (* n * = 8).

The OSCILLATOR system was developed and optimised for Arabidopsis. To test if the system can be used for other species without major modification 32 day old plants of two additional model species; petunia(Figure 5a) and tomato (Figure 5b) were analysed. In its vegetative stage petunia has a rosette structure (Figure 5a) and therefore growth analysis could be measured using OSCILLATOR without any modification. Figure 5c shows the projected leaf length measured for the petunia plants. From these curves the projected oscillations were extracted (Figure 5d). Tomato plants displayed strong circumnutations (variable apex position in time). This made tracking of the central meristem necessary for calculation of the projected distance. After correction for centre displacement, clear diurnal rhythms in leaf growth movement could be extracted (Figure 5d).Both petunia (V26) and tomato (Money Maker) displayed a phase of 18.0 ± 0.27 and 18.3 ± 1.21 hours respectively, which is about two hours later than the phase of Arabidopsis (*e.g*. 16.1 ± 0.21 hr. for Col-0). Results indicate that each species displays a unique leaf growth movement pattern which can be considered the integrated result of the effects of light, temperature and the endogenous circadian oscillator on plant development.

## Conclusions

The natural variation we identified within six Arabidopsis accessions matched previous described observations, demonstrating the validity of our system. Variation among natural accessions has been studied under continuous light conditions before [5,27]. OSCILLATOR now provides the possibility to study leaf growth and movement under various diurnal conditions, which more closely mimic the natural environment. Characterisation of leaf growth movement by phase, period and amplitude allows easy comparison between different genotypes and treatments. Our system also opens up the perhaps even more intriguing possibility to evaluate plant behaviour under gradual changing photoperiods (mimicking seasonal transitions). Diurnal rhythms in leaf growth and movement are directly related to plant growth and help repositioning of leaves relative to the light and could contribute to increased photosynthetic capacities [22,28,29]. Furthermore, leaf growth movement and related changes in rosette compactness have been shown to facilitate cooling and allow adaptation to increasing ambient temperatures [30]. Combined, the results show that OSCILLATOR can be used to extract parameters of leaf growth movement which can be used to characterise different genotypes. OSCILLATOR provides plant scientists with a relatively cheap, reliable and non-invasive tool to accurately dissect diurnal growth rhythms of various plant species under continuing day/night cycles.

## Methods

### Plant material and growth conditions

Seeds of *Arabidopsis thaliana* accessions were provided by M. van Zanten (Laboratory of Molecular Plant Physiology, Utrecht University, The Netherlands) and J. Keurentjes (Laboratory of Plant Genetics, Wageningen University, The Netherlands). Seeds of *Petunia hybrida *(v26) were donated by Tom Gerats (Laboratory of Plant Genetics, Radboud University Nijmegen, The Netherlands). Seeds of tomato (*S. lycopersicum* L.) cultivar; Money Maker were obtained from Wouter Kohlen (Max Planck Institute for Plant Breeding Research, Köln, Germany). All experiments were performed in automated climate controlled WEISS ( http://www.wkt.com) cabinets (12/12 hours light/dark cycle). Relative humidity was kept constant at 60% (v/v) and photosynthetic active radiation (PAR) was 150 μmol m^2^ s^-1^ from white fluorescents tubes (PHILIPS, type T5, Colour code: 840). Ambient temperature cycles for growth were 22°C (photoperiod) and 12°C (dark period) with a temperature ramp of 0.33°C/min. Measurements showed that soil temperature lagged ~20 minutes behind ambient air temperature. Plants were grown in fertilised peat / perlite based soil in square (5x5x5 cm) plastic pots with different genotypes placed at random positions in the growth cabinet. Plants were placed on an irrigation mat which was watered automatically to saturation through porous tubing from a basin containing tap-water every three days at the start of the photoperiod. After 20 days plants were watered once with half strength Hoagland-nutrient solution instead of water. Five days later plants were transferred to a second climate cabinet for imaging with similar conditions and an IR camera system with IR lights. Plants were allowed to acclimate for seven days before the onset of imaging. Thus, at the start of imaging plants were 32 days old and the Arabidopsis accessions all had 13–14 true leaves.

### Plant growth imaging and image data analysis

The pipeline for imaging and image analysis as used by OSCILLATOR is summarised in figure S4. To enable automated leaf tip tracking the 11^th^ and 12^th^ real leaf was marked with inert paint before the start of imaging although for manual tip tracking this is not necessary (tip tracking will be explained in more detail later). The 11^th^ and 12^th^ leaf were analysed and the leaf length varied from 5–8 mm. Imaging was with SLR NIKON D90 digital camera’s with a NIKON AF 50 mm F/1.8 lens. Cameras were powered by net adaptors ( http://www.nikon.com). To enable night photography, cameras were custom modified by MAXMAX ( http://www.maxmax.com) for removal of the internal IR filter to allow detection of IR light. Sufficient IR illumination per cabinet was provided by two continuous burning LED lights (890 nm, 12 W, KÖNIG electronics, ( http://www.konigelectronic.com). Each camera was connected to a dedicated laptop with active USB 2.0 repeater cables and controlled with time-lapse photography software (NIKON Camera Control Pro 2; http://www.nikon.com). Camera settings were fixed; F/stop = f/8, Exposure time = 1/5 sec., ISO speed = ISO-200. Field of view for each camera was 16 rosette plants. Photographs were taken every 20 minutes and stored as individual images. Sequential images were imported as virtual stacks into ImageJ ( http://rsbweb.nih.gov/ij/) and the image stack was subsequently cropped into individual plant image stacks. For cropping of single plant image stacks, the desired areas can be selected in the first frame and cropping of all the stacks is further automatic (Additional file 4: Figure S4b). The resulting multiple single plant ‘virtual stacks’ were manually saved as individual plant image stacks and named as appropriate. Each individual plant image stack was used for leaf tip tracking, using either the manual tracking (Additional file 5: File S1, http://rsbweb.nih.gov/ij/plugins/track/track.html) or the automated MOSAIC particle tracking plugin (Additional file 5: File S1, http://www.mosaic.ethz.ch/Downloads/ParticleTracker). The manual tracking plugin allows semi-automated selection of leaf tip coordinates throughout the stack (Additional file 4: Figure S4c). To facilitate this procedure Standard Mouse Auto Clicker 2.8 (Additional file 5: File S1) can be used to automate screen mouse clicks at specified intervals and any location on the screen. Alternatively, when selected leaves are marked with a small paint dot at the start of the experiment, automated tracking of the leaf tip with the particle tracker MOZAIC plugin can be used. However, the particle recognition occasionally fails in single frames, resulting in gaps in the leaf tip tracks. This then requires manual correction, which can be labour intensive. Therefore, in this work we used the manual tracking plugin. In combination with Standard Mouse Auto Clicker 2.8 (Additional file 5: File S1) set at 1 click per 0.2 second a typical stack of 500 images is manually processed in 100 seconds. Both the manual and automated tracking plugins provide output format compatible with MICROSOFT EXCEL 2010 ( http://office.microsoft.com) which can be individually named and saved by the user for each individual leaf track (*e.g.* Plant1.1.xls etc.; Additional file 4: Figure S4d).

### Extraction of parameters of leaf growth oscillations with the OSCILLATOR script

To determine the relative leaf movement, for each image the distance between leaf tip and plant centre needs to be calculated. In our experiments the Arabidopsis and petunia rosette centres were static throughout the image stack and thus were provided by the single manually determined (x/y) coordinates of the rosette apical centre. For tomato the apex positions varied throughout the stack and apex position was determined using ‘manual tracking plugin’ (Additional file 5: File S1). The leaf tip and apex coordinates of all individual leaves of a single genotype (2 leaves per plant, 4 plants per genotype) are subsequently combined in the OSCILLATOR_input.csv file (Additional file 5: File S1, Additional file 4: Figure S4e). For each genotype, the OSCILLATOR_input.csv file was placed in designated folders each containing the OSCILLATOR SCRIPT (Additional file 5: File S1, Additional file 4: Figure S4f). For each folder the script was activated in R 2.13.1 ( http://www.r-project.org/), which generates the following outputs:

### Projected length for individual leaves and averaged projected length

The distance between the leaf tip and the plant apex was calculated by the OSCILLATOR.R script based on the following equation:

$$\text{Distance}=\sqrt{{\left(x_{2}-x_{1}\right)}^{2}+{\left(y_{2}-y_{1}\right)}^{2}\text{.}}$$

The resulting distance (projected leaf length) is provided for single leaves in output file project_length.csv. In addition the average is plotted against time as a graph with SE depicted as shading (Projectedlength.jpeg).

### Raw projected oscillations for individual leaves and averaged raw projected oscillations

Subsequently a best fit second degree polynomial trend line is automatically calculated for each individual projected length curve and the residual values are subtracted from this line. The resulting residuals were inverted to allow maximum upright leaf position to correspond to maximum peak height. These raw projected oscillations are provided as data in output file AvgRawOscillation.csv. In addition the average is plotted against time with SE as shading in AvgRawOscillations.jpeg (Additional file 4: Figure S4g).

### Smoothed projected oscillations for individual leaves and smoothed averaged projected oscillations

The raw oscillations of individual leaves are then smoothed using WAVECLOCK script [21] imbedded in the OSCILLATOR script (Additional file 5: File S1). The smoothed projected oscillations are provided for single leaves in the file IndividualSmoothedOscillation.csv and as an average with SE in the file AverageSmoothedOscillations.csv. In addition the average is plotted against time with SE depicted as shading in AvgSmoothedOscillation.jpeg (Additional file 4: Figure S4g).

### Phase and amplitude of individual leaves and averaged phase, amplitudes and period

The peak values for the smoothed projected oscillations of individual leaves for all periods are used to calculate the phase and amplitude. The phase and amplitude data are provided for single leaves in file phaseDays.csv and amplitudeDays.csv respectively and are then averaged and plotted against time with SE in graph RplotphaseMinMax.jpeg and RplotampMinMax.jpeg respectively (Additional file 4: Figure S4g). Period information is plotted as a wavelet scalogram (Wavelet_1.png) [21].

Given all materials and software in place it will take an experienced user approximately one hour to complete a single genotype set (four plants, eight leaves) analysed throughout a stack of imaging representing seven days of development as typically performed in this study. All data presented is the typical result of at least two independent experiments, each based on at least 7 individual leaf track analysis.

### Statistical analyses

Statistical differences between accessions were determined using one-way ANOVA. Individual differences were then identified using a post-hoc Tukey test (P < 0.05). All analysis were performed using SAS_9.20 ( http://www.sas.com/).

## Competing interests

The authors declare that they have no competing interests.

## Authors’ contributions

The experiments were planned by RB and AvdK. RB performed the experiments, analysed the data and prepared the figures. MM constructed the OSCILLATOR. R script. The manuscript was written by RB, AvdK and HB. All authors read and approved the final manuscript.

## Supplementary Material

Additional file 1 **Figure S1.** Validation experiments. (a) Additional IR light does not affect leaf growth movement of Col-0. SE are depicted as shading ( * n=8 * ). (b) Leaf angles were calculated from the absolute leaf lengths and their phase corresponds to the phase of the smoothed projected oscillations of Col-0 ( * n = 8 *) (c) Col-0 smoothed projected oscillations increase gradually and decrease again during development. The red block show the timeframe during which all further experiments were performed, ( * n = 8 *). (d) Final projected leaf lengths were plotted against the averaged amplitudes of individual leaves of 6 different accessions, ( * n * =8 for all accessions except for Cvi-1,* n = 7 *). Click here for file

Additional file 2 **Figure S2.** Natural variation in initial petiole angle as previously reported [22]**.** Values represent absolute angles (degrees) relative to the horizontal of Arabidopsis accessions measured at a fixed time point [22]. Black bars indicate selected accessions screened with OSCILLATOR (Adapted and reproduced with permission). Error bars represent SE ( * n * (petioles) * ≥ 8 *). Click here for file

Additional file 3 **Figure S3.** Timing of peak oscillations (phase) for selected accessions depicted per period (day). Grey colour indicates night. For all accessions * n=8 * leaves except for Cvi-0 ( * n=7 * ). Error bars represent SE. Click here for file

Additional file 4 **Figure S4.** Schematic representation of the different steps of OSCILLATOR. (a) Hardware consisting of cameras which are connected to a dedicated laptop are controlled by Nikon camera control software. (b) Sequential images were imported as virtual stacks into ImageJ. For cropping of single plant slices the desired area needs to be selected in the first frame and cropping is automatic throughout the stack. The resulting single plant ‘virtual stacks’ were then saved as ‘image sequence’. (c) Leaf tip tracking was performed with the manual tracking plugin which allows semi-automated selection of leaf tip coordinates throughout the stack. Alternatively, if selected leaves are marked with a small paint dot at the start of the experiment this allows the particle tracker MOZAIC plugin to track the dot throughout the virtual image stack. Both plugins are provided in File S1. (d) The output of the leaf tip tracking plugins is provided as MICROSOFT EXCEL files containing the X and Y values for each image (frame) which can be named and saved as appropriate. (e) Centre coordinates are determined for each plant (Xc, Yc) and are combined with the leaf tip track coordinates (X,Y) in the input file (OSCILLATOR input.csv). (f) The OSCILLATOR input.csv file is placed in a dedicated folder together with the OSCILLATOR SCRIPT. This folder directory is set in R software and the OSCILLATOR script is run (source(“OSCILLATOR.R”)). (g) As the script runs output files are provided in the folder containing the script and input file. For each step the data is provided for individual leaves and as average including SE. In addition these averages (±SE) are plotted against time and provided as JPEG files. Click here for file

Additional file 5 **File S1.** OSCILLATOR package: Plugins, scripts and example file. All plugins and scripts needed for OSCILLATOR are provided and combined in File S1.zip. All the components of this file are described in more detail in Figure S4 and the methods section. In addition an example input file (OSCILLATOR_input) is included. Plugins needed for image processing in ImageJ and Standard Mouse Auto Clicker 2.8 are freeware and have been included for completeness but were not developed by the authors. Click here for file
