# Supplementary material for: OSCILLATOR: A system for analysis of diurnal leaf growth using infrared photography combined with wavelet transformation
Source: Plant Methods. 2012 Aug 7;8:29. doi: 10.1186/1746-4811-8-29 (PMC3489599; doi:10.1186/1746-4811-8-29)
Supplement: Additional file 1 — Figure S1. Validation experiments. (a) Additional IR light does not affect leaf growth movement of Col-0. SE are depicted as shading ( n=8 ). (b) Leaf angles were calculated from the absolute leaf lengths and their phase corresponds to the phase of the smoothed projected oscillations of Col-0 ( n = 8 ) (c) Col-0 smoothed projected oscillations increase gradually and decrease again during development. The red block show the timeframe during which all further experiments were performed, ( n = 8 ). (d) Final projected leaf lengths were plotted against the averaged amplitudes of individual leaves of 6 different accessions, ( n =8 for all accessions except for Cvi-1, n = 7 ). [file 1746-4811-8-29-S1.pdf]

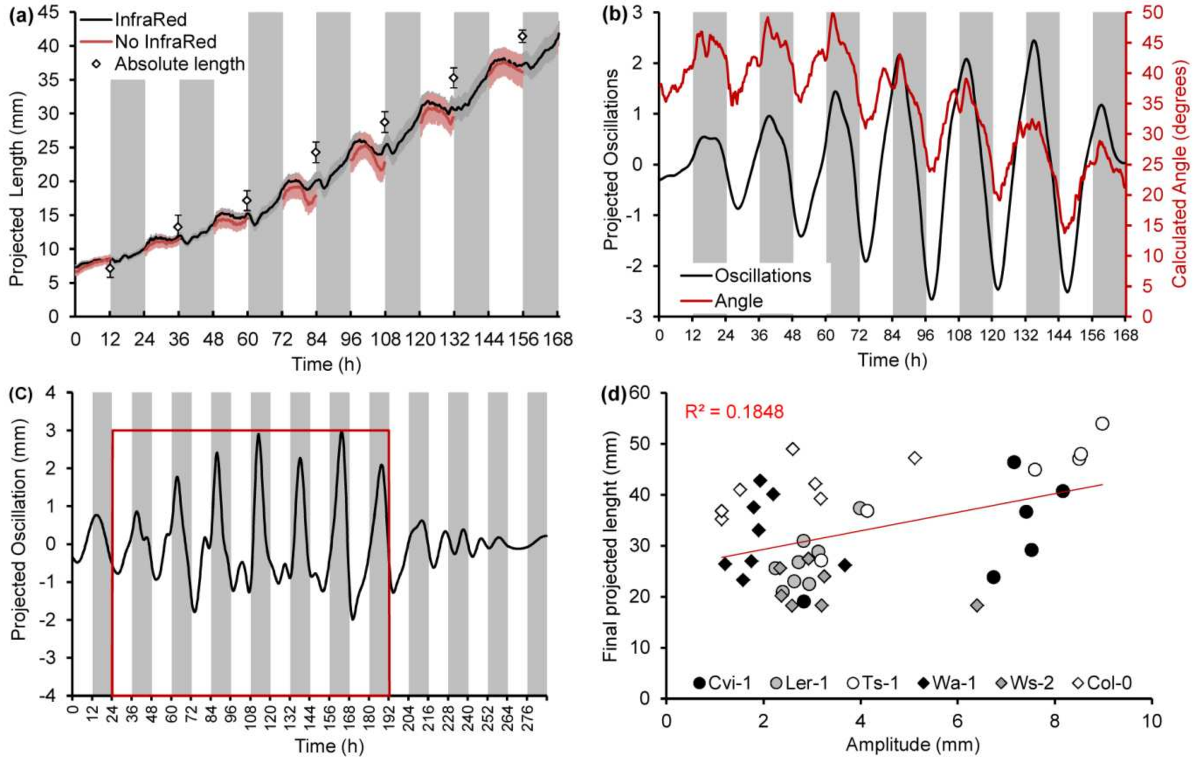

Figure S1. Validation experiments.

(a) Additional IR light does not affect leaf growth movement of Col-0. SE are depicted as shading (*n=8*).

(b) Leaf angles were calculated from the absolute leaf lengths and the phase of calculated angles corresponds to the phase of the smoothed projected oscillations of Col-0 (*n=8*).

(c) Col-0 smoothed projected oscillations increase gradually and decrease again during development. The red block show the timeframe during which all further experiments were performed. (*n=8*).

(d) Final projected leaf lengths were plotted against the averaged amplitudes of individual leaves of 6 different accessions. (*n* =8 for all accessions except for Cvi-1 *n=7*).
