# Supplementary material for: OSCILLATOR: A system for analysis of diurnal leaf growth using infrared photography combined with wavelet transformation
Source: Plant Methods. 2012 Aug 7;8:29. doi: 10.1186/1746-4811-8-29 (PMC3489599; doi:10.1186/1746-4811-8-29)
Supplement: Additional file 2 — Figure S2. Natural variation in initial petiole angle as previously reported [22]. Values represent absolute angles (degrees) relative to the horizontal of Arabidopsis accessions measured at a fixed time point [22]. Black bars indicate selected accessions screened with OSCILLATOR (Adapted and reproduced with permission). Error bars represent SE ( n (petioles) ≥ 8 ). [file 1746-4811-8-29-S2.pdf]

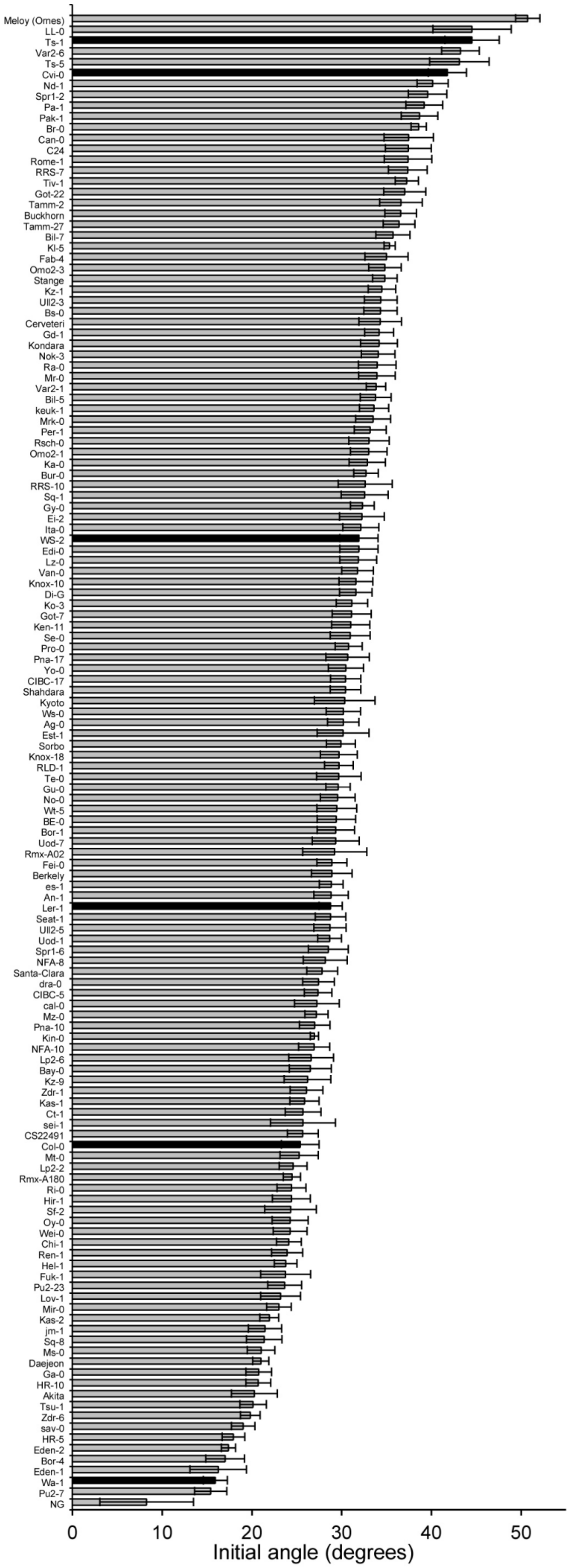

Figure S2

Natural variation in initial petiole angle as previously reported [22].

Values represent absolute angles (degrees) relative to the horizontal of Arabidopsis accessions measured at a fixed time point [22]. Black bars indicate selected accessions screened with OSCILLATOR. Error bars present SE (n (petioles) ≥ 8). (Adapted and reproduced with permission).
