# Supplementary material for: OSCILLATOR: A system for analysis of diurnal leaf growth using infrared photography combined with wavelet transformation
Source: Plant Methods. 2012 Aug 7;8:29. doi: 10.1186/1746-4811-8-29 (PMC3489599; doi:10.1186/1746-4811-8-29)
Supplement: Additional file 3 — Figure S3. Timing of peak oscillations (phase) for selected accessions depicted per period (day). Grey colour indicates night. For all accessions n=8 leaves except for Cvi-0 ( n=7 ). Error bars represent SE. [file 1746-4811-8-29-S3.pdf]

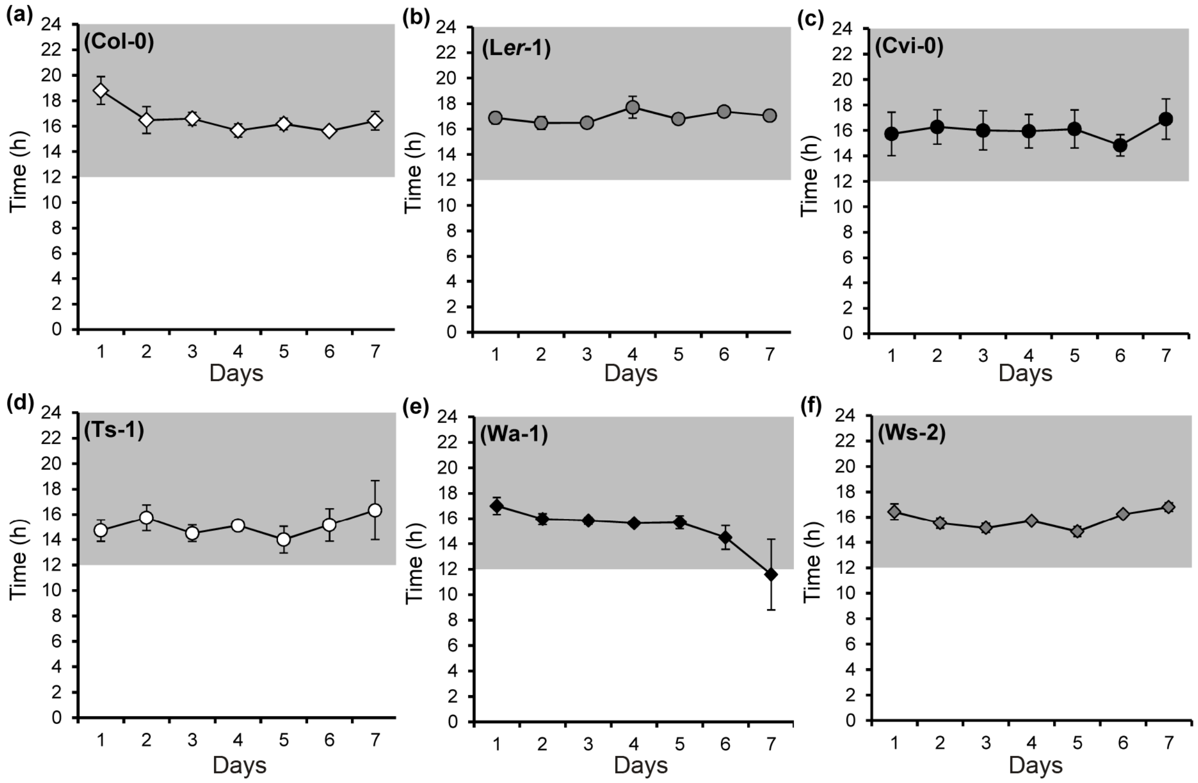

Figure S3. Timing of peak oscillations (phase) for selected accessions depicted per day.

Grey colour indicates night. For all accessions *n=8* leaves except for Cvi-0 (*n=7*). Error bars represent SE.
