# Supplementary material for: OSCILLATOR: A system for analysis of diurnal leaf growth using infrared photography combined with wavelet transformation
Source: Plant Methods. 2012 Aug 7;8:29. doi: 10.1186/1746-4811-8-29 (PMC3489599; doi:10.1186/1746-4811-8-29)
Supplement: Additional file 4 — Figure S4. Schematic representation of the different steps of OSCILLATOR. (a) Hardware consisting of cameras which are connected to a dedicated laptop are controlled by Nikon camera control software. (b) Sequential images were imported as virtual stacks into ImageJ. For cropping of single plant slices the desired area needs to be selected in the first frame and cropping is automatic throughout the stack. The resulting single plant ‘virtual stacks’ were then saved as ‘image sequence’. (c) Leaf tip tracking was performed with the manual tracking plugin which allows semi-automated selection of leaf tip coordinates throughout the stack. Alternatively, if selected leaves are marked with a small paint dot at the start of the experiment this allows the particle tracker MOZAIC plugin to track the dot throughout the virtual image stack. Both plugins are provided in File S1. (d) The output of the leaf tip tracking plugins is provided as MICROSOFT EXCEL files containing the X and Y values for each image (frame) which can be named and saved as appropriate. (e) Centre coordinates are determined for each plant (Xc, Yc) and are combined with the leaf tip track coordinates (X,Y) in the input file (OSCILLATOR input.csv). (f) The OSCILLATOR input.csv file is placed in a dedicated folder together with the OSCILLATOR SCRIPT. This folder directory is set in R software and the OSCILLATOR script is run (source(“OSCILLATOR.R”)). (g) As the script runs output files are provided in the folder containing the script and input file. For each step the data is provided for individual leaves and as average including SE. In addition these averages (±SE) are plotted against time and provided as JPEG files. [file 1746-4811-8-29-S4.pdf]

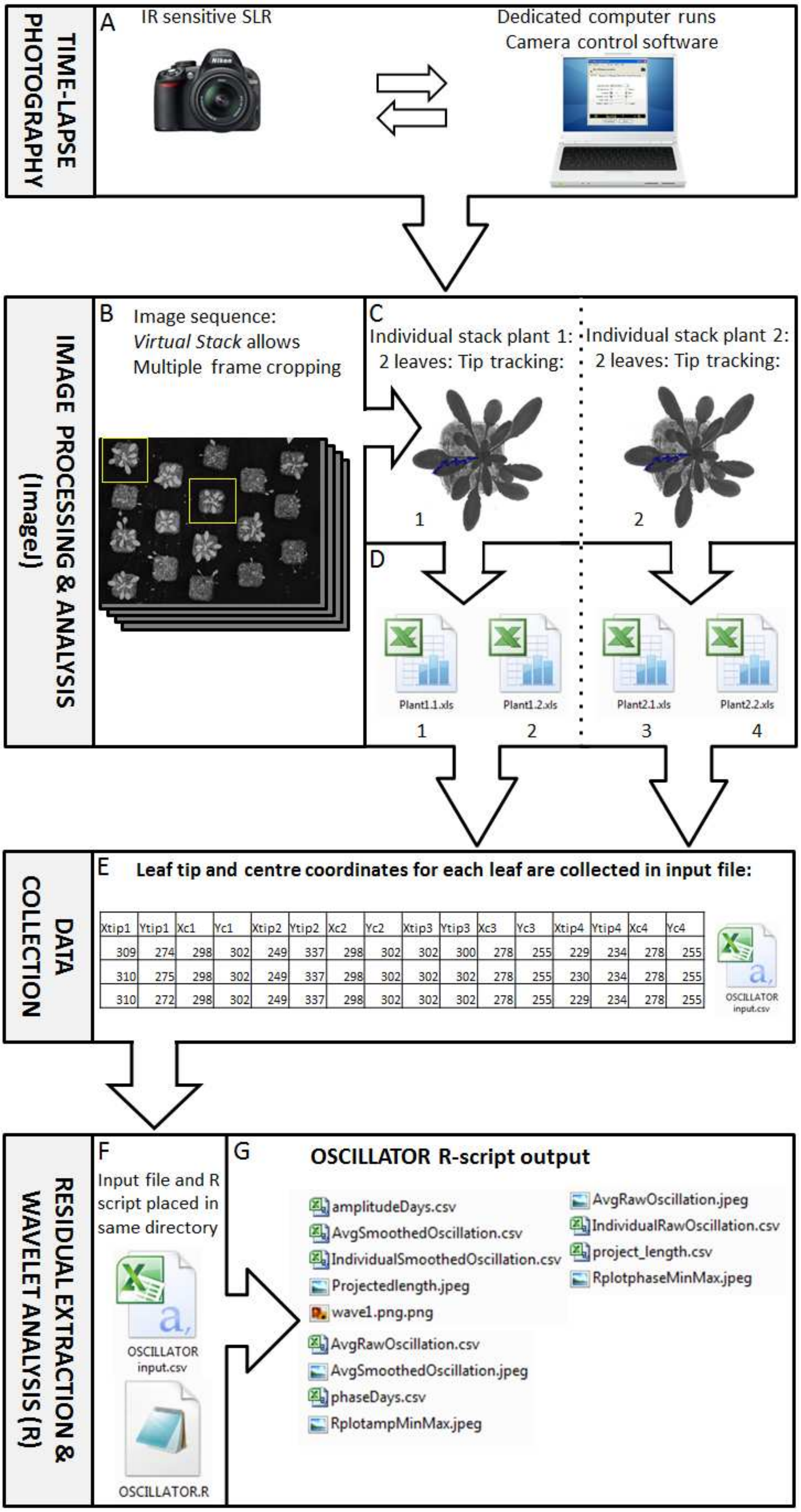
TIME-LAPSE PHOTOGRAPHY
A
IR sensitive SLR
Dedicated computer runs Camera control software
IMAGE PROCESSING & ANALYSIS (ImageJ)
B
Image sequence: *Virtual Stack* allows Multiple frame cropping
C
Individual stack plant 1: 2 leaves: Tip tracking:
1
Individual stack plant 2: 2 leaves: Tip tracking:
2
D
Plant1.1.xls
1
Plant1.2.xls
2
Plant2.1.xls
3
Plant2.2.xls
4
DATA COLLECTION
E
Leaf tip and centre coordinates for each leaf are collected in input file:

Xtip1	Ytip1	Xc1	Yc1	Xtip2	Ytip2	Xc2	Yc2	Xtip3	Ytip3	Xc3	Yc3	Xtip4	Ytip4	Xc4	Yc4
309	274	298	302	249	337	298	302	302	300	278	255	229	234	278	255
310	275	298	302	249	337	298	302	302	302	278	255	230	234	278	255
310	272	298	302	249	337	298	302	302	302	278	255	229	234	278	255

OSCILLATOR input.csv
RESIDUAL EXTRACTION & WAVELET ANALYSIS (R)
F
Input file and R script placed in same directory
OSCILLATOR input.csv
OSCILLATOR.R
G
OSCILLATOR R-script output
amplitudeDays.csv
AvgSmoothedOscillation.csv
IndividualSmoothedOscillation.csv
Projectedlength.jpeg
wave1.png.png
AvgRawOscillation.csv
AvgSmoothedOscillation.jpeg
phaseDays.csv
RplotampMinMax.jpeg
AvgRawOscillation.jpeg
IndividualRawOscillation.csv
project_length.csv
RplotphaseMinMax.jpeg

Figure S4. Schematic representation of the different steps of OSCILLATOR.

(a) Hardware consists of a IR sensitive camera which is connected to a dedicated laptop and controlled by Nikon camera control software.

(b) Sequential images of the plant matrix were imported as virtual stacks into ImageJ. For cropping of single plant stacks the desired area needs to be selected in the first frame and cropping is automatic throughout the stack. In this example 2 plants are cropped resulting in 2 single plant ‘virtual stacks’ which were then saved as ‘image sequence’.

(c) Leaf tip tracking was performed with the manual tracking plugin which allows semi-automated selection of leaf tip co-ordinates throughout the stack. For each plant, 2 leaves are tracked resulting in 2 sets of tip-coordinates per stack. Alternatively, if selected leafs are marked with a small paint dot at the start of the experiment this allows the particle tracker MOZAIC plugin to track the dot throughout the virtual image stack (File S1).

(d) The output of the leaf tip tracking plugins is provided as MICROSOFT EXCEL files containing the X and Y values for each image (frame) of a single plant stack. This file can be named and saved as appropriate (*e.g.* Plant1.1). In this example 2 leafs of 2 plants were tracked resulting in 4 output files.

(e) Centre coordinates are determined for each plant (Xc, Yc) and are combined with the leaf tip track coordinates (X,Y) of all leaves in the input file (OSCILLATOR input.csv).

(f) The OSCILLATOR input.csv file is placed in a designated folder together with the OSCILLATOR SCRIPT. This folder directory is set in R software and the OSCILLATOR script is run (source(“OSCILLATOR.R”)).

(g) As the script runs output files are provided in the folder containing the script and input file. For each step the data is provided for individual leaves and averaged including SE. In addition these averages (±SE) are plotted against time and provided as graphs (.JPEG).
